# Operative Efficiency, Technical Workflow, and Perioperative Outcomes in Minimally Invasive Trans-Kambin Oblique Thoracic Interbody Fusion (MIS-OTIF): A Retrospective Clinical Analysis

**DOI:** 10.7759/cureus.114281

**Published:** 2026-08-10

**Authors:** Hamid Abbasi, Abdul Rauf, Dominic VanKadlen

**Affiliations:** 1 Spine Surgery, Avicenna Technical University, Burnsville, USA; 2 Neurosurgery, Inspired Spine, Minneapolis, USA

**Keywords:** electromyography (emg), interbody fusion, kambin triangle, mis-otif, neuromonitoring, oblique thoracic interbody fusion, thoracic disc herniation, thoracic spine surgery, thoracolumbar junction, trans-kambin approach

## Abstract

Background

Thoracolumbar fusion surgery has historically been associated with prolonged operative duration, extensive posterior muscle dissection, elevated blood loss, prolonged hospitalization, and increased perioperative morbidity. Over the last decade, minimally invasive spinal surgery has evolved substantially with the goal of reducing tissue disruption while preserving biomechanical stabilization and neural decompression. Minimally invasive trans-Kambin oblique thoracic interbody fusion (MIS-OTIF) represents an extension of minimally invasive principles into the thoracic and thoracolumbar spine through an oblique trans-Kambin corridor that minimizes posterior soft tissue injury while facilitating efficient instrumentation and interbody reconstruction.

Objective

To evaluate operative efficiency, perioperative workflow characteristics, obesity-related technical considerations, and complication profiles in patients undergoing MIS-OTIF procedures.

Methods

A retrospective review of consecutive patients undergoing MIS-OTIF procedures between 2020 and 2026 was performed. Variables analyzed included operative time, body mass index (BMI), revision status, number of instrumented levels, hybrid versus pure minimally invasive technique, and complications.

Results

Twenty-five MIS-OTIF procedures were reviewed. Operative duration ranged from 30 to 171 minutes depending on procedural complexity, revision status, and the addition of posterior instrumentation or open components. Mean BMI across the cohort demonstrated that MIS-OTIF remained technically feasible even in obese and morbidly obese patients. Single-level thoracic MIS-OTIF procedures demonstrated the shortest operative duration, while hybrid constructs, multilevel fusion procedures, and revision surgeries required longer operative times. Elevated BMI alone did not significantly increase operative duration. Complication rates remained low, with isolated cases of persistent stenosis identified which were unrelated to surgical technique itself.

Conclusion

MIS-OTIF demonstrates favorable operative efficiency with acceptable operative duration across a broad range of thoracic and thoracolumbar pathologies, including obese and revision patients. The minimally invasive trans-Kambin oblique corridor may reduce operative exposure time, tissue disruption, blood loss, and physiologic stress while maintaining efficient workflow and stabilization. Larger multicenter prospective studies are warranted to further validate the reproducibility and long-term outcomes of MIS-OTIF.

## Introduction

Thoracolumbar pathology presents significant technical challenges due to the unique biomechanical and anatomical characteristics of the thoracic spine, thoracolumbar junction, and upper lumbar region. Traditional open thoracolumbar fusion techniques often require extensive muscular dissection, prolonged operative exposure, and substantial posterior soft tissue disruption. These factors may contribute to increased blood loss, prolonged operative duration, postoperative pain, infection risk, delayed mobilization, and increased perioperative morbidity [1-3].

Minimally invasive spine surgery (MISS) has transformed modern spinal surgery through reduced tissue disruption and smaller incisions. These techniques have consistently led to improved perioperative recovery, reduced hospitalization, and lower blood loss. Although minimally invasive lumbar interbody fusion techniques have been extensively studied, relatively limited literature exists regarding minimally invasive thoracic and thoracolumbar fusion strategies [4-6].

Minimally invasive oblique thoracic interbody fusion (MIS-OTIF) utilizes an oblique extraforaminal trans-Kambin posterolateral corridor to access the thoracic and thoracolumbar spine while minimizing posterior muscular disruption. The approach is designed to remain outside the pleural cavity, thereby avoiding routine violation of the pleural space and potentially reducing approach-related thoracic morbidity compared with transthoracic techniques. Through fluoroscopic guidance, targeted access, tubular or expandable retractors, and percutaneous instrumentation, MIS-OTIF may provide several perioperative advantages, including reduced muscular dissection, decreased blood loss, faster surgical exposure, shorter operative duration, reduced postoperative pain, earlier mobilization, and improved workflow efficiency [6-9].

The clinical viability of trans-Kambin access trajectories relies heavily on specialized tubular systems designed to bypass standard open surgery and expand narrow bone corridors safely [10,11]. The thoracolumbar junction represents a biomechanically demanding region transitioning from the rigid thoracic spine to the mobile lumbar spine [11]. Surgical intervention in this region frequently involves patients with obesity, multilevel degeneration, adjacent segment disease, deformity, stenosis, or prior instrumentation. Operative efficiency becomes increasingly important in these complex patients due to anesthesia duration, infection risk, blood loss, surgeon fatigue, and overall perioperative morbidity [12,13].

The purpose of this study was to evaluate operative efficiency and perioperative workflow characteristics of MIS-OTIF in a retrospective consecutive series while examining the impact of obesity, multilevel constructs, revision surgery, and hybrid procedures on operative duration.

The thoracic spine differs substantially from the lumbar spine in terms of pedicle morphology, spinal canal dimensions, rib articulations, kyphotic alignment, and neural vulnerability. The thoracic spinal cord occupies a greater proportion of the spinal canal than in the lumbar region, thereby increasing the potential neurologic risk associated with instrumentation or canal violation [12,14].

The thoracolumbar junction (T10-L2) represents a transition zone between thoracic kyphosis and lumbar lordosis. This region experiences substantial biomechanical stress and is particularly vulnerable to degenerative instability, adjacent segment disease, compression fractures, junctional failure, progressive stenosis, and deformity progression [12,15].

Pedicle anatomy in the thoracic spine demonstrates considerable variability. Upper thoracic pedicles are smaller and more medially angulated, increasing technical complexity during instrumentation placement. Fluoroscopic visualization may also become challenging due to rib overlap, shoulder obstruction, obesity, and osteopenia [14,16]. Image quality and visualization may present a challenge as well due overlapping of lung and abdominal tissue density differences.

Important anatomical considerations during MIS-OTIF include preservation of the thoracic spinal cord, exiting nerve roots, dorsal root ganglia, segmental vessels, and radicular arteries. Potential neurologic complications include pedicle breach, dural injury, spinal cord compression, root injury, intercostal neuralgia, and neuromuscular complications and pneumothorax [10,14].

The minimally invasive oblique trajectory may reduce posterior muscular disruption while preserving posterior tension band integrity and limiting exposure-related morbidity.

## Materials and methods

Study design

A retrospective review of consecutive patients undergoing MIS-OTIF procedures between 2020 and 2026 was performed.

Inclusion criteria

Patients were included in the study if they underwent MIS-OTIF for symptomatic thoracic or thoracolumbar pathology located between T6 and L1 after failure of appropriate conservative management for at least four to six months.

Eligible patients demonstrated clinical symptoms and radiographic findings consistent with neural compression, instability, or degenerative thoracolumbar disease requiring surgical decompression and stabilization. Surgical indications included thoracic disc herniation, thoracic disc disease, symptomatic foraminal or central canal stenosis, degenerative disc disease, adjacent segment degeneration, thoracolumbar instability, recurrent or residual stenosis, pseudarthrosis, and mechanical axial back pain associated with radiographic instability.

Diagnosis and surgical candidacy were established through correlation of patient symptoms, neurologic examination, advanced imaging studies, and failure of nonoperative treatment including medications, physical therapy, spinal injections, or activity modification. In selected cases, provocative discography or diagnostic spinal injections were utilized to further confirm the symptomatic level and pain generator.

Both primary and revision thoracolumbar fusion procedures were included, including single-level, multilevel, hybrid minimally invasive, and reinstrumentation cases, consistent with accepted thoracolumbar surgical indications.

Exclusion criteria

Patients with severe spinal deformity, including fixed scoliosis, marked hyperkyphosis, or complex congenital vertebral anomalies, were excluded because these conditions significantly alter normal thoracic anatomical landmarks, pedicle orientation, rib articulation, and sagittal alignment, thereby limiting safe access through the minimally invasive oblique thoracic corridor. Such deformities may increase the technical difficulty of fluoroscopic localization, reduce safe working angles for tubular access and instrumentation, and elevate the risk of neural, vascular, or instrumentation-related complications. Pathology extending above the T6 vertebral level was also excluded because upper thoracic anatomy presents additional technical limitations, including smaller pedicle dimensions, greater spinal cord occupancy within the canal, interference from the scapula and shoulder girdle during fluoroscopic visualization, restricted operative corridors related to rib cage anatomy, and limited instrument maneuverability. These anatomical constraints may compromise safe MIS-OTIF access, visualization, decompression, and instrumentation accuracy. 

Variables collected

Variables analyzed included operative duration, fluoroscopy time, body mass index (BMI), surgical approach and procedure type, number of operative levels treated, revision surgery status, hybrid versus fully minimally invasive surgical technique, perioperative and postoperative complications, reinstrumentation requirements, need for open posterior supplemental fixation, estimated blood loss (EBL), and hospital length of stay.

Operative time analysis

Operative time was defined as total operative duration from incision to closure. Cases were subdivided into fully MIS-OTIF procedures, hybrid procedures involving open posterior supplementation, and revision procedures involving prior instrumentation or decompression.

BMI stratification

Patients were categorized according to adult BMI ranges published by the Centers for Disease Control and Prevention (CDC). BMI was calculated as weight in kilograms divided by height in meters squared (kg/m²). Patients were classified as underweight (BMI <18.5 kg/m²), healthy weight (BMI 18.5-24.9 kg/m²), overweight (BMI 25.0-29.9 kg/m²), Class I obesity (BMI 30.0-34.9 kg/m²), Class II obesity (BMI 35.0-39.9 kg/m²), and Class III obesity (BMI ≥40.0 kg/m²) [7].

Statistical analysis

Continuous variables were reported as mean ± standard deviation. Descriptive subgroup comparisons were performed between pure minimally invasive surgery versus hybrid procedures, obese versus non-obese patients, single-level versus multilevel constructs, and primary versus revision surgery.

Human subjects

Consent for treatment and publication was obtained or waived by all participants included in this study. Ethical review and approval for this study were provided by Pearl pathways institution review board (IRB; approval No.2025-0186), which reviewed the submitted study materials and determined that the research qualified for exempt status under applicable federal regulations governing human subjects research. Specifically, the study was determined to be exempt under 45 CFR 46.104(d)(4)(ii) and 45 CFR 46.104(d)(4)(iii) for Secondary Research Uses of Identifiable Private Information or Identifiable Biospecimens. The study was conducted in accordance with institutional requirements and applicable ethical standards.

Surgical technique and instrumentation

**Figure 1:**
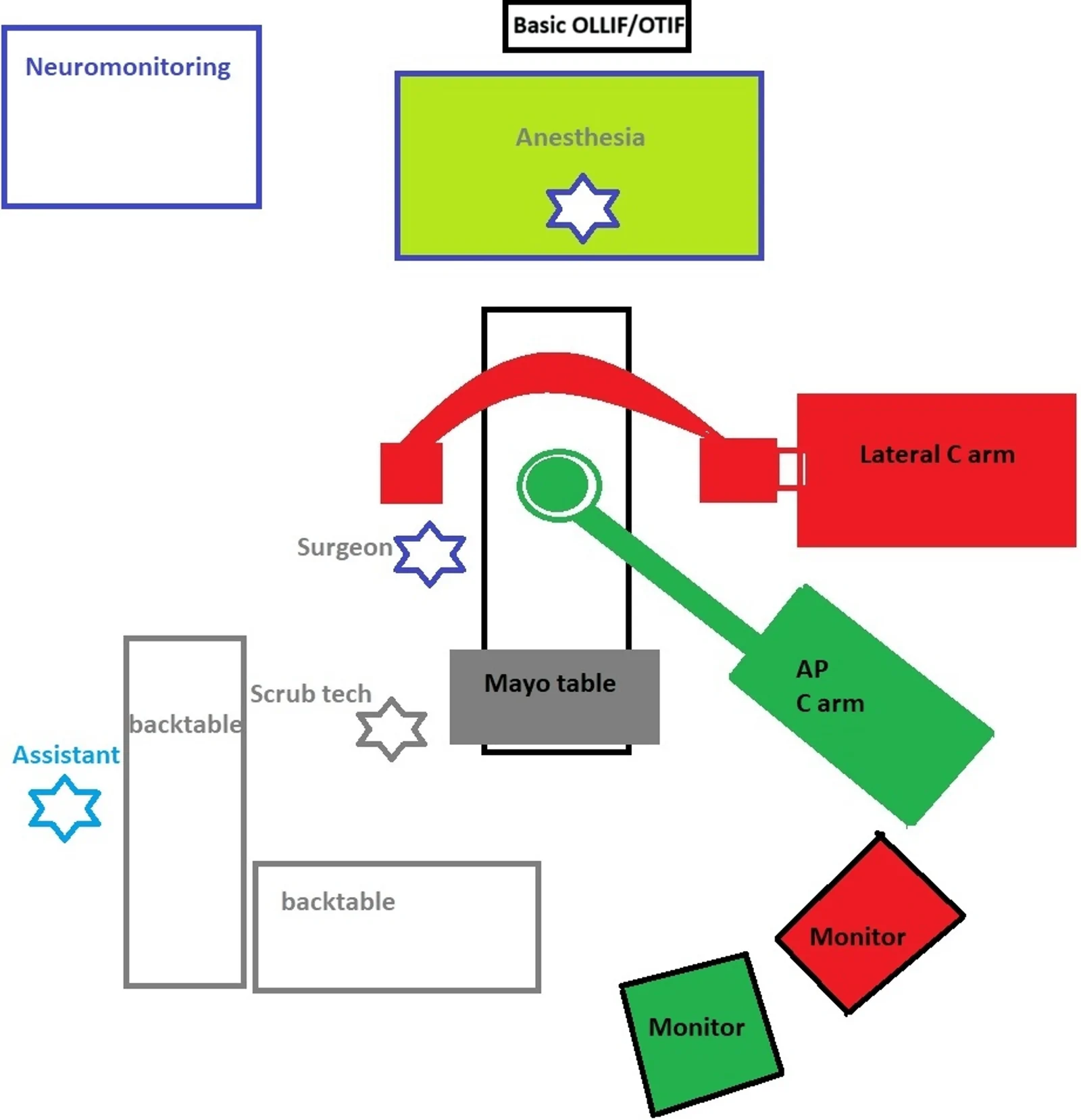
Operative setup for the procedure This was identical to the standard oblique lateral lumbar interbody fusion (OLLIF) positioning, demonstrating the patient in a prone position on a radiolucent table with appropriate fluoroscopic alignment for intraoperative guidance. OTIF: oblique thoracic interbody fusion; AP C-arm: anterior-osterior C-arm. Image credits: Created by Abbasi H. using Microsoft Paint (Microsoft Corp., Redmond, WA, USA) and edited using Adobe Photoshop (Adobe Inc., San Jose, US).

Patients were positioned prone on a radiolucent operating table with meticulous padding of all pressure points to minimize perioperative neuropathy and optimize fluoroscopic visualization. Accurate thoracic and thoracolumbar level localization was confirmed using biplanar fluoroscopy prior to incision planning [15,16]. The overall operating room setup, including patient positioning, fluoroscope orientation, and instrumentation arrangement, was standardized following principles similar to the oblique lateral lumbar interbody fusion (OLLIF) technique, as illustrated in Figure 1, which demonstrates the stepwise setup and intraoperative configuration.

Instrumentation

Instrumentation was performed using minimally invasive spinal systems designed to facilitate percutaneous access, interbody preparation, and posterior stabilization. The implant systems utilized included the PathLoc-L MIS system (LnK Biomed, Seoul, South Korea), the Zeus-O system (Amendia, Inc., Marietta, GA, USA), or the OLLIF system (Advanced Research Medical, Burnsville, MN, USA). These systems provided the necessary instrumentation for establishing a controlled trans-Kambin working corridor, performing interbody fusion, and achieving stable fixation while maintaining the minimally invasive principles of MIS-OTIF.

Surgical planning and trajectory

**Figure 2:**
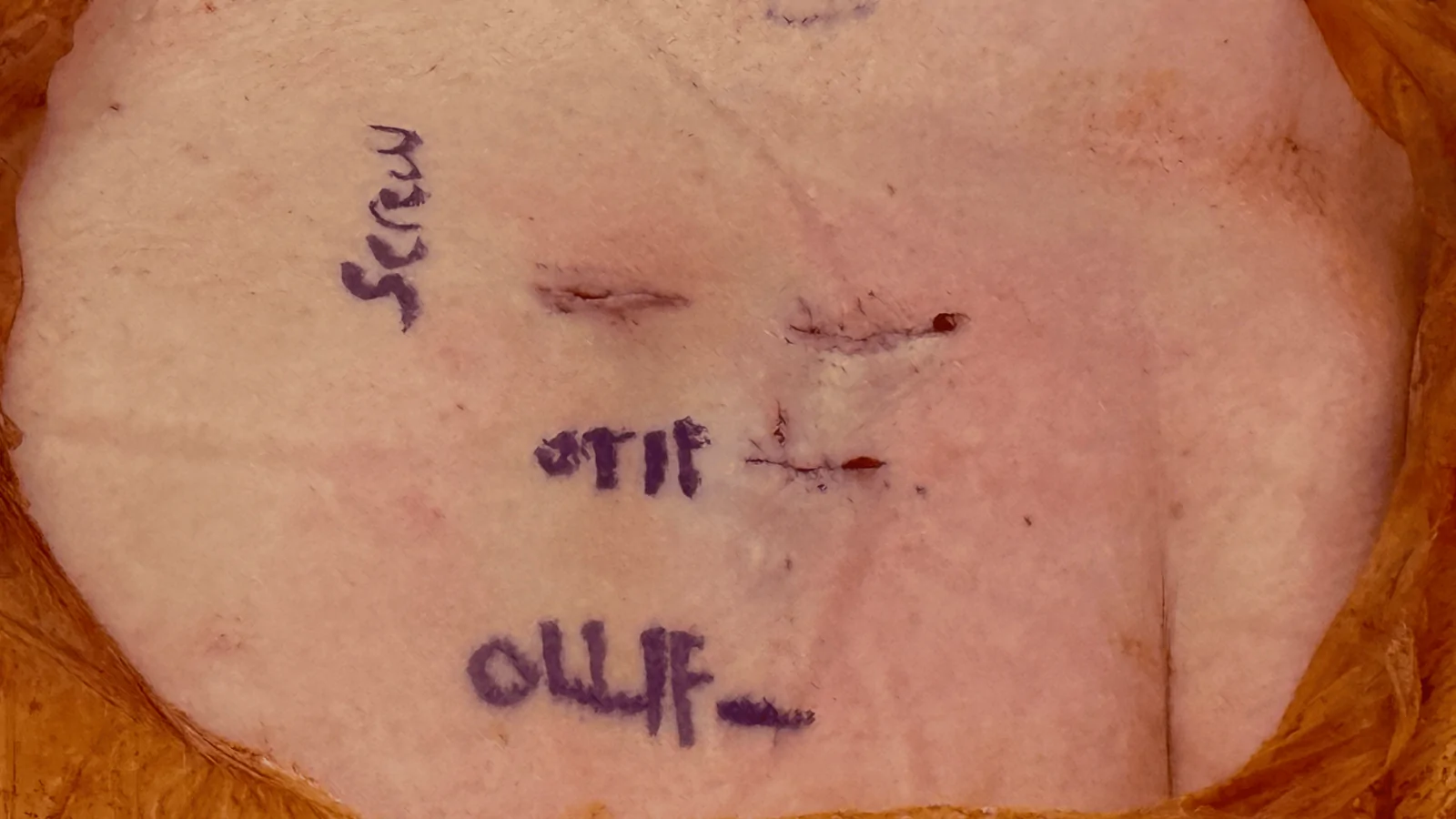
Comparison of skin incision placement in OLLIF and OTIF techniques This demonstrates the difference in entry levels and incision positioning on the patient’s back. OLLIF: oblique lateral lumbar interbody fusion; OTIF: oblique thoracic interbody fusion.

The MIS-OTIF approach differs substantially from standard lumbar OLLIF because of thoracic anatomical constraints including rib articulation, pleural proximity, scapular obstruction, narrower disc spaces, reduced foraminal dimensions, and close proximity of major vascular structures. In lumbar OLLIF procedures, there is typically a greater lateral distance between pedicle screw placement, skin incision, and cage insertion, allowing multiple cages to be placed through a single incision. In contrast, MIS-OTIF requires a more medialized and steeper trajectory due to the restrictive thoracic rib cage anatomy and limited working corridor (Figure 2).

Prior to surgery, the target disc space was confirmed on both anteroposterior and lateral fluoroscopic views by counting the levels from both cranial and caudal reference points and correlating them with the ribs. These intraoperative findings were also cross-referenced with the preoperative CT scan, which included the entire thoracolumbar region.

This step was essential because accurate localization of the pathologic segment in the thoracolumbar spine can be extremely challenging. Contributing factors include frequent anatomic variation, transitional vertebral levels, limited intraoperative imaging quality at the thoracoabdominal junction, differences in tissue density between the thoracic and abdominal regions, and image-averaging artifacts encountered with C-arm fluoroscopy.

Skin incision planning was therefore more segment-specific in MIS-OTIF. Based on patient habitus, a 2 cm paramedian 7-11 cm from midline incision was made directly over the target thoracic level just above the rib of the same level under fluoroscopic guidance, aligned with the intended disc space trajectory rather than fixed surface landmarks. The incision was tailored to allow a direct trans-Kambin posterolateral corridor while avoiding rib head obstruction and minimizing the risk of pleural violation. Because of the segmental angulation of the thoracic spine and reduced working corridor length, each treated level often required an independent incision, particularly in multilevel constructs where cage placement cannot be safely achieved through a single access portal.

**Figure 3:**
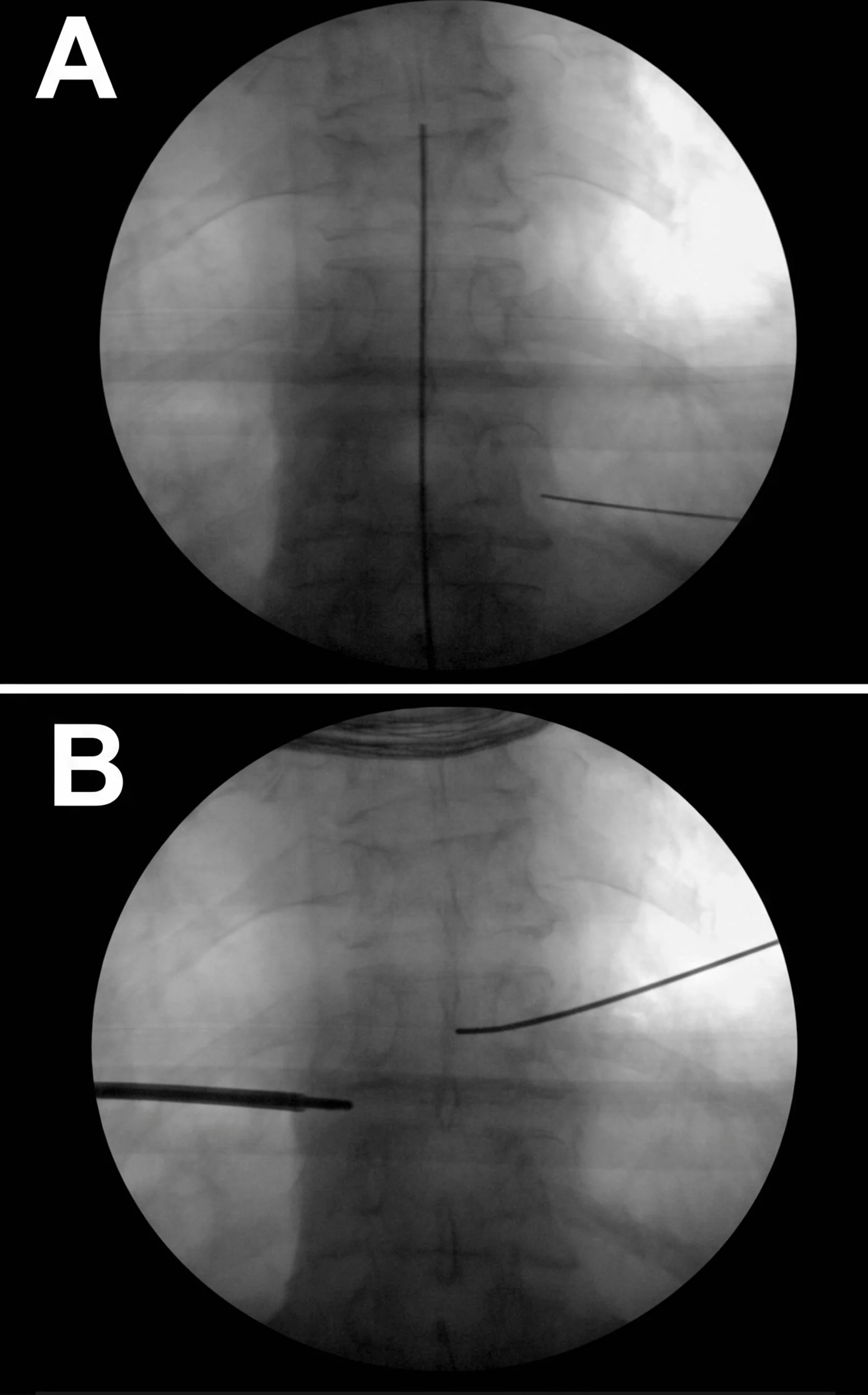
Fluoroscopic identification of the midline and safe probe trajectory through Kambin's triangle (A) Fluoroscopic identification of the radiographic anatomical midline demonstrating alignment of the spinous processes between the pedicles for accurate trajectory planning. Spinal needles were positioned over the pedicles to serve as radiographic markers; (B) Illustration of the safe probe trajectory through Kambin's triangle, demonstrating the optimal corridor for interbody disc access while avoiding injury to the exiting and traversing nerve roots.

Under fluoroscopic guidance, the anteroposterior (AP) view was optimized by rotating and adjusting the fluoroscope until the spinous processes were centered between the pedicles and the target disc space was clearly visualized. The fluoroscopic crosshair was then aligned directly over the center of the target disc space along the radiographic midline. Establishing a true AP view was critical for accurate trajectory planning, ensuring symmetric access to the disc space while minimizing the risk of mal positioned instrumentation, asymmetric cage placement, or neural injury (Figure 3).

In contrast to lumbar OLLIF, where the entry point is commonly positioned at approximately one-half of the disc depth from the middle if the disc in AP and lateral view, the MIS-OTIF entry point was established closer to one-third of the disc depth on the AP fluoroscopic view and approximately two-thirds disc depth on the lateral fluoroscopic view. This modified trajectory allowed safer access to the thoracic disc space while accommodating the narrower anatomical corridor and surrounding thoracic structures.

**Figure 4:**
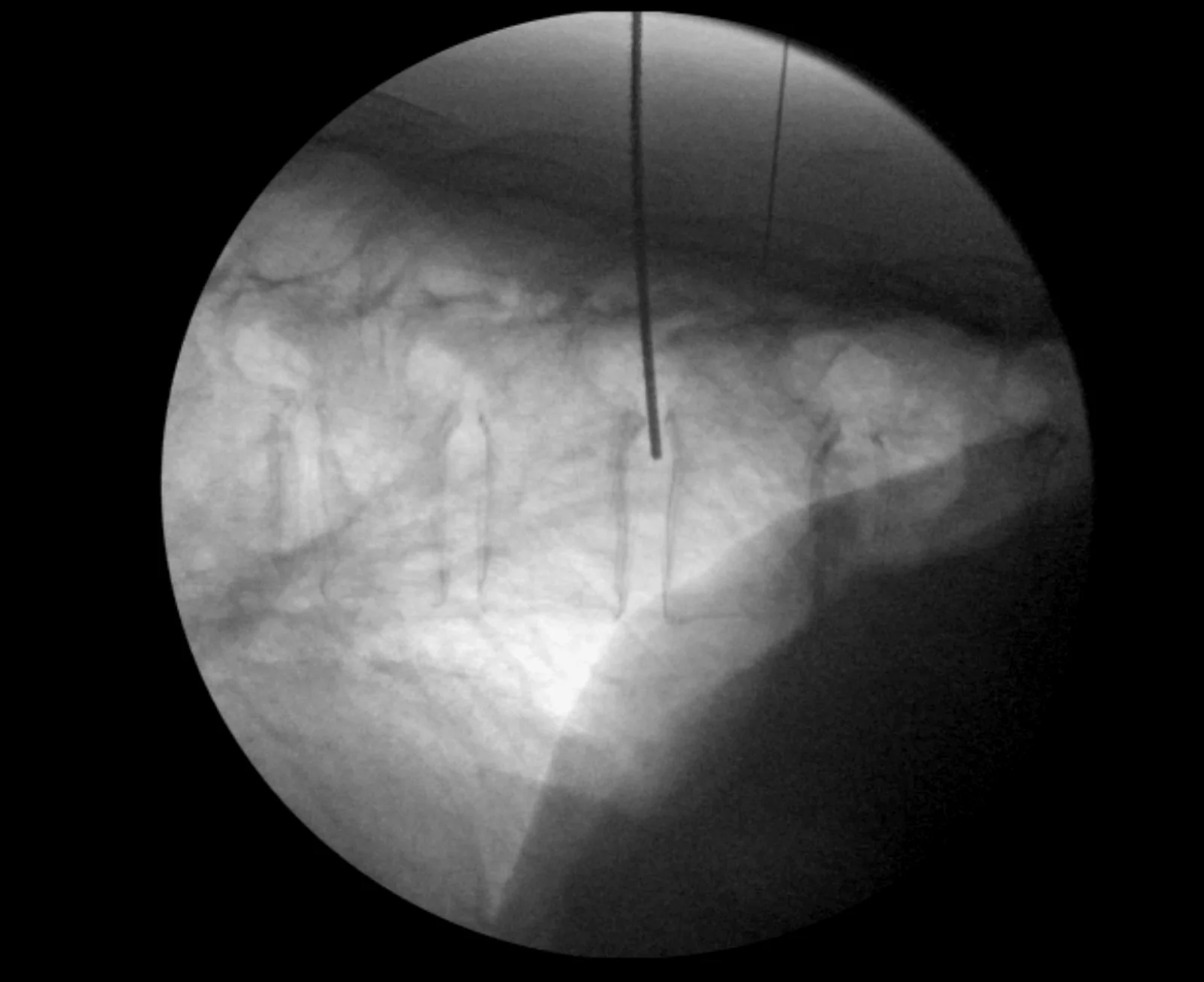
Lateral fluoroscopic view demonstrating depth visualization showing lateral trajectory to the disc at the appropriate level to guide safe and controlled access to the intervertebral disc space

Figure 4 shows the lateral fluoroscopic view demonstrating probe trajectory and depth during advancement, showing the entry point positioned at the appropriate disc depth to guide safe and controlled access to the intervertebral disc space.

 This modified entry point was necessary to safely navigate over the rib prominence while preserving a posterior extra pleural corridor. Positioning the trajectory too lateral increased the risk of rib obstruction and pleural violation, whereas excessively medial positioning increased the risk of spinal canal violation and proximity to the thoracic aorta. As a matter of fact, lateral approach and entry to pleural cavity has been described previously in our paper describing a different technique, MIS-DTIF (direct thoracic interbody fusion) [17].

**Figure 5:**
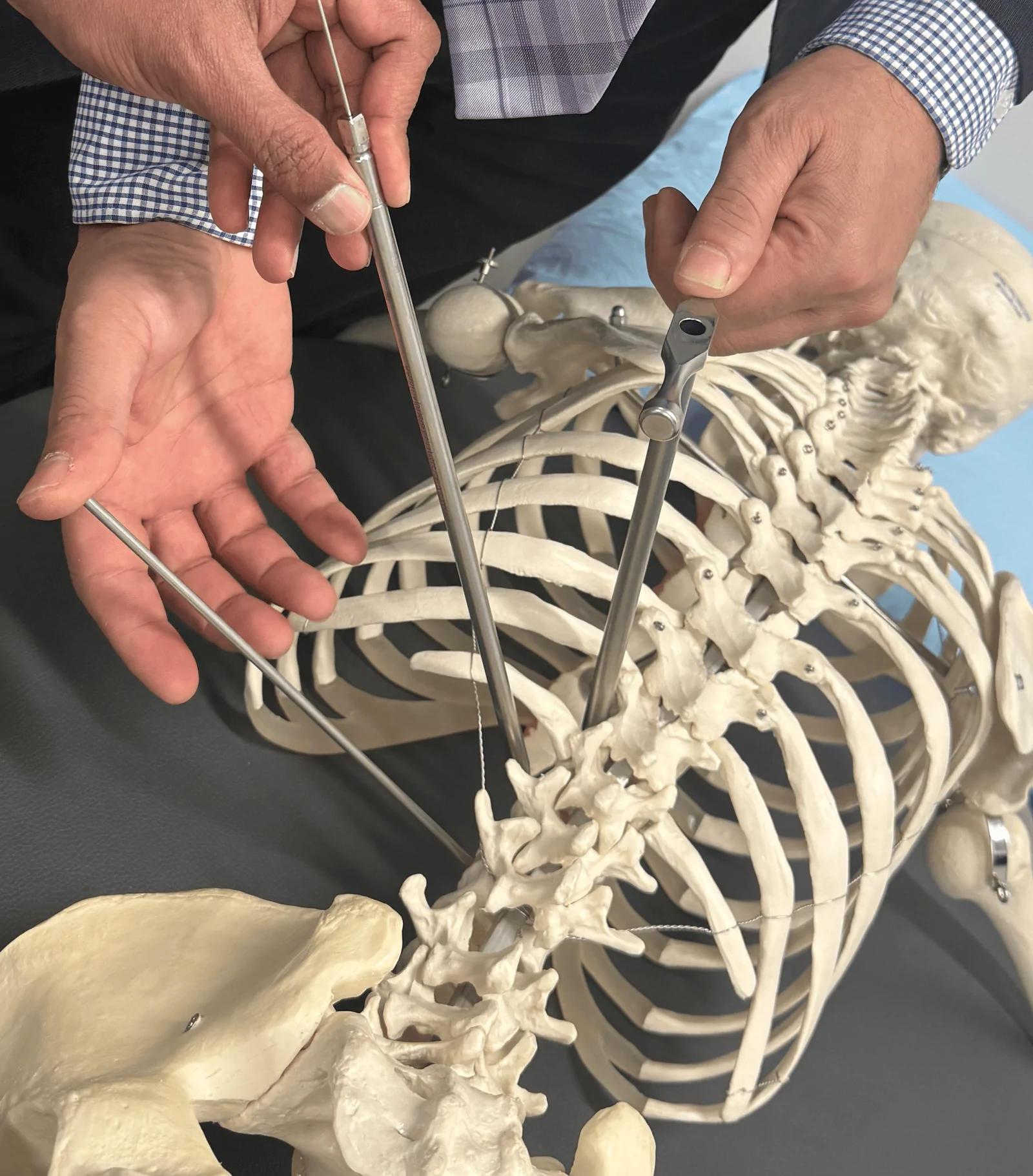
Comparison of approach angles in MIS-OTIF, OLLIF, and DLIF Illustration demonstrates the differences in surgical trajectories and approach angles among minimally invasive trans-Kambin oblique thoracic interbody fusion (MIS-OTIF), oblique lateral lumbar interbody fusion (OLLIF), and direct lateral interbody fusion (DLIF).

The approach angle in MIS-OTIF was substantially steeper than standard lumbar OLLIF approaches, typically approximately 30 degrees from the vertical axis or roughly 60 degrees from the horizontal plane (Figure 5).

This steep oblique angle enabled true trans-Kambin access to the thoracic disc space while remaining posterior to the pleural cavity and lateral to critical neural structures. In comparison the angle of approach in OLLIF is 40-50 and MIS-DTIF is 25-40 degrees [18].

Rib-based extrapleural access technique

Throughout the procedure, triggered stimulation of the probe was performed at 3 mA, the probe was insulated with Teflon coating, concentrating the electrical current at the exposed metallic tip.

Before incision, neuromonitoring electrodes were placed in the intercostal and abdominal muscle groups to detect any electromyographic activity corresponding to the segmental innervation of the thoracic nerve root of interest. This allowed continuous monitoring for nerve root irritation or proximity during probe advancement in the thoracic region. 

The rib immediately superior to the target disc space was identified fluoroscopically and served as the principal anatomical guide throughout access development. A small, approximately 2 cm paramedian, incision was made just above the rib. 

Maintaining uninterrupted osseous contact during probe advancement was a critical safety maneuver because it prevented uncontrolled advancement through soft tissues and minimized the risk of pleural violation, lung puncture, vascular injury, or pneumothorax. The rib-guided trajectory also provided a reproducible anatomical corridor for safe thoracic access.

Steps for the trans-Kambin OTIF approach

**Figure 6:**
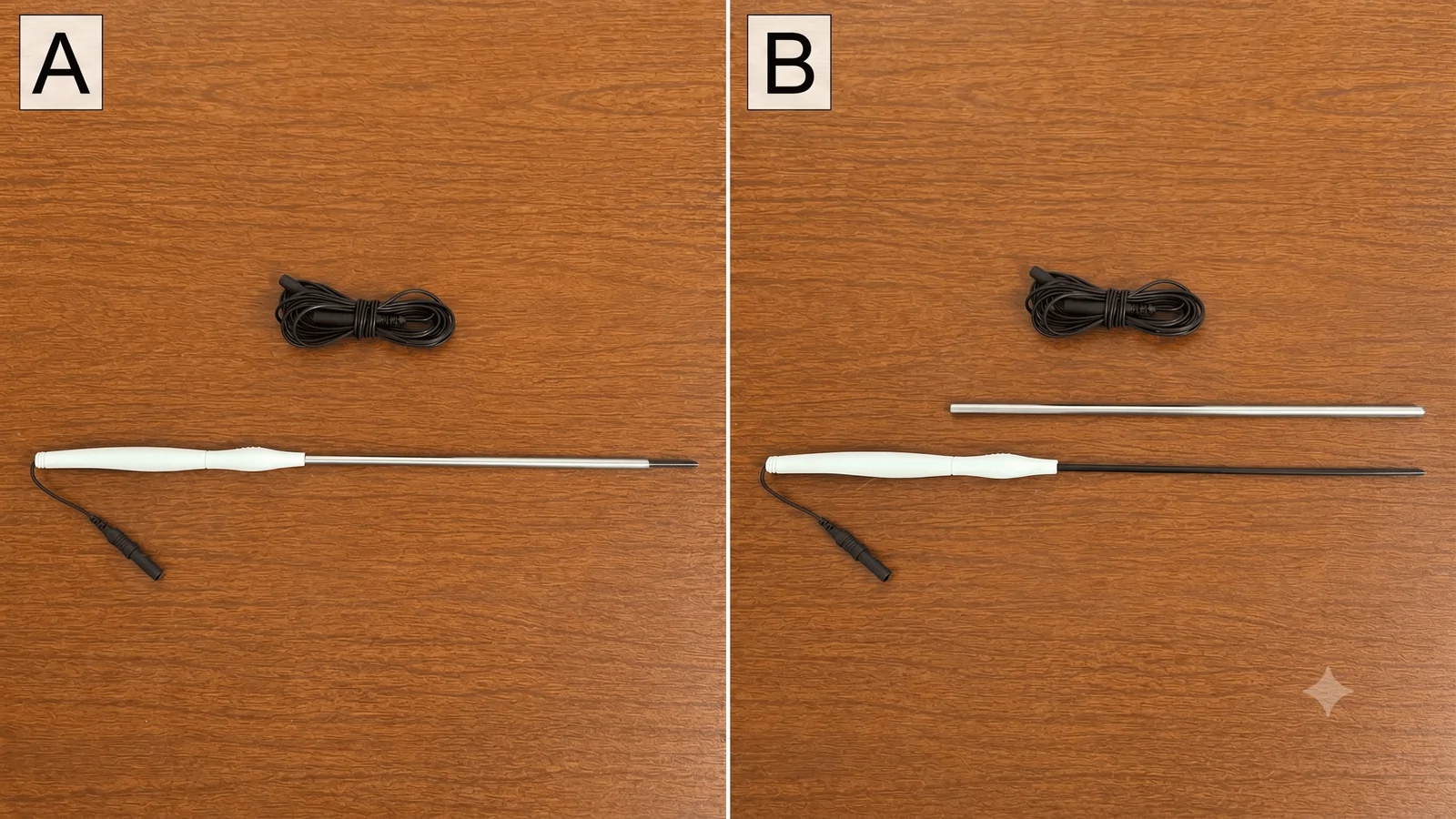
Teflon-coated neuromonitoring probe and trajectory sleeve (A) Teflon-coated neuromonitoring probe with the trajectory sleeve assembly used during minimally invasive trans-Kambin oblique thoracic interbody fusion (MIS-OTIF) access. The probe is advanced through the trans-Kambin triangle under real-time electromyographic (EMG) monitoring to identify and avoid adjacent neural structures while establishing a safe trajectory toward the target disc space. Once the disc space is safely reached, the trajectory sleeve is positioned to maintain the working corridor, allowing subsequent passage of the guide wire and sequential dilators; (B) Separate components of the neuromonitoring probe and trajectory sleeve demonstrating the relationship between the stimulation probe and access sleeve used during MIS-OTIF preparation.

After appropriate preoperative measurements and confirmation of the entry point, a cannulated, Teflon-coated neuromonitoring probe (Figure 6) was introduced under continuous stimulation at 3 mA.

The probe was insulated along its shaft, with only the distal tip exposed. A 1 inch shorter metal sleeve was over the probe to keep the trajectory once the probe is removed later.

The probe is advanced beneath the skin and carefully walked along the upper part of rib toward the rib head. Once the rib head is reached, the probe is directed into the Kambin triangle. At this stage, stimulation is increased to 4 mA to confirm an adequate safety margin from the exiting nerve root. If no electromyographic response is obtained at 4 mA, the metal sleeve is advanced over the probe and seated on the disc capsule.

The neuromonitoring probe is then removed, sleeve keeps the trajectory, then a sharp-tipped K-wire is advanced through the sleeve into the disc space under biplanar fluoroscopic guidance. The metal sleeve is subsequently removed, leaving the K-wire in place as the established trajectory from the skin to the disc space.

An 8-mm cannulated dilator with smooth edges is then passed over the K-wire. The dilator is advanced gently through the soft tissues and then carefully tapped into the disc space, maintaining the fluoroscopically confirmed trajectory throughout the maneuver.

A working tube was then advanced over the probe into the disc space, thereby creating a protected minimally invasive working corridor from the skin surface directly into the thoracic interbody space. The surrounding skin, intercostal musculature, and soft tissues maintained a relatively sealed access tract throughout the procedure, which helped minimize pleural air communication even if pleural space is accidentally violated and reduced the likelihood of postoperative pneumothorax.

**Figure 7:**
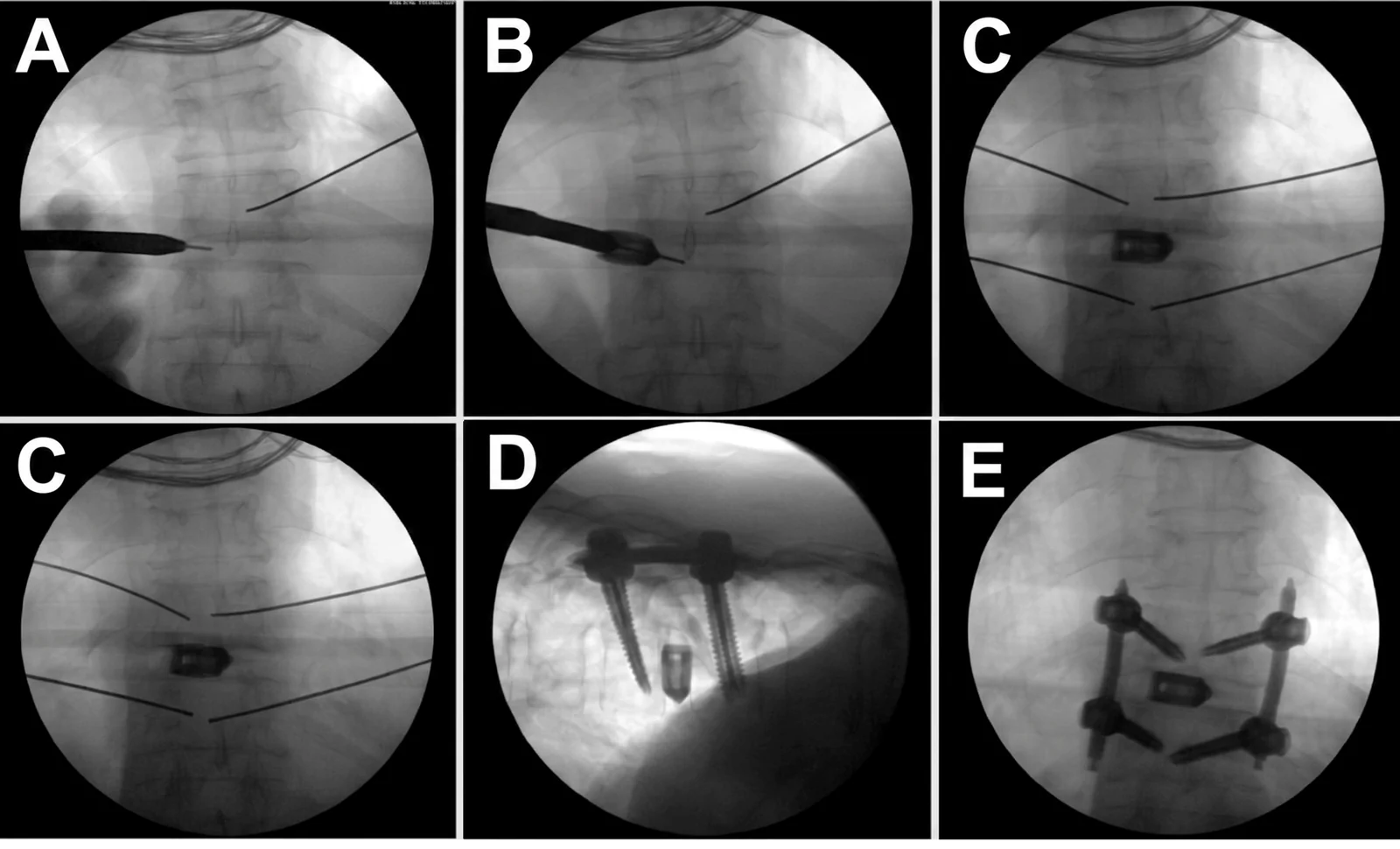
Intraoperative fluoroscopic sequence during minimally invasive trans-Kambin oblique thoracic interbody fusion (MIS-OTIF) (A) Anteroposterior (AP) fluoroscopic view demonstrating advancement of the dilator into the disc space along the planned trajectory, confirming appropriate positioning and controlled soft-tissue dilation before definitive instrument placement; (B) AP fluoroscopic view demonstrating delivery and positioning of the interbody cage within the disc space along the planned trajectory, confirming accurate trajectory before final cage placement; (C) AP fluoroscopic view demonstrating pedicular K-wire placement and final interbody cage position within the disc space, confirming maintenance of trajectory alignment and appropriate implant positioning before pedicle screw fixation; (D) Lateral fluoroscopic view demonstrating the final position of the interbody cage and pedicle screw construct, confirming appropriate implant placement, restoration of disc height, and sagittal alignment; (E) AP fluoroscopic view demonstrating the final position of the interbody cage and pedicle screw construct, confirming appropriate coronal alignment, construct symmetry, and satisfactory implant placement.

The sequential stages of the MIS-OTIF procedure under fluoroscopic guidance are illustrated in Figure 7.

Figure 7 demonstrates the progression from initial disc access and controlled soft-tissue dilation to interbody cage insertion, pedicular K-wire placement, and final construct assessment on anteroposterior and lateral fluoroscopic views. Collectively, they highlight accurate trajectory development, precise implant positioning, maintenance of spinal alignment, and successful completion of interbody fusion with posterior pedicle screw fixation.

Thoracic trans-Kambin approach

MIS-OTIF utilized a true thoracic trans-Kambin approach. Sequential dilation and tubular retractors enabled targeted exposure while preserving paraspinal musculature and minimizing posterior muscular stripping. The oblique thoracic corridor facilitated direct disc access while preserving posterior tension band integrity and limiting exposure-related blood loss [6,9].

Because thoracic spinal procedures involve close proximity to the spinal cord and relatively narrow canal dimensions, heightened emphasis was placed on meticulous fluoroscopic guidance, tactile feedback, and continuous anatomical orientation throughout the procedure. Instrument advancement was performed using a strictly bone-referenced technique. Under continuous fluoroscopy, instruments deliberately “slid” along the pedicle, rib head, and vertebral body rather than advancing freely through soft tissue planes. This approach constrained instrument trajectory using fixed osseous landmarks and reduced the risk of neural injury, pleural violation, vascular injury, or mal positioned instrumentation [9,16].

Decompression and interbody fusion

Following physiologic decompression, discectomy and endplate preparation were performed under fluoroscopic visualization. Interbody cages were then inserted through the oblique thoracic corridor followed by percutaneous pedicle screw and rod fixation using minimally invasive instrumentation techniques [5,16,18].

The steep thoracic approach angle imposed limitations on implant size and biologic graft volume. Compared with lumbar OLLIF procedures, smaller cages were often required because of restricted thoracic working angles, limited disc space dimensions, and rib-related constraints. Similarly, the amount of biological material that could be delivered through the working channel was smaller compared to trans-Kambin OLLIF.

Safety considerations and risk avoidance

Several important safety considerations were emphasized throughout the procedure. Remaining posterior to the pleural cavity substantially reduced the risk of pneumothorax compared with direct lateral thoracic approaches. The technique also avoided the need for costotransversectomy, thereby reducing tissue disruption and operative morbidity associated with more extensive open thoracic exposure.

Because the approach remained medial to the scapular border, MIS-OTIF could also be utilized in upper thoracic levels where MIS-DTIF approaches are frequently limited by scapular obstruction.

Careful awareness of anterior vascular anatomy was essential, particularly near the thoracolumbar junction where the thoracic aorta lies in close proximity to the vertebral bodies. Biplanar fluoroscopy is extremely helpful to avoid excessively medial instrumentation or aggressive anterior disc preparation which could increase the risk of neural or vascular injury.

At completion of the procedure, instruments were removed sequentially and the access tract was immediately packed with sponge material to minimize pleural air entry and ensure secure soft tissue closure. Layered closure was then performed through minimal-access incisions while preserving surrounding musculature and soft tissue integrity.

## Results

Cohort characteristics

A total of 25 patients underwent MIS-OTIF between September 2019 and February 2026. Patient demographic and procedural characteristics are summarized in Table 1. 

**Table 1:** Patient demographics and operative outcomes Continuous variables are presented as Mean ± Standard Deviation (SD), and categorical variables are presented as Count (%).

Variable	Value (Mean ± SD) or Count (%)
Total patients	25
Mean age (years)	61.0 ± 12.6
Sex	
Male	13 (52.0%)
Female	12 (48.0%)
Mean BMI (kg/m²)	33.9 ± 7.1
Severe obesity (BMI ≥40 kg/m²)	6 (24.0%)
Procedure level	
Single-level procedures	10 (40.0%)
Multilevel procedures	15 (60.0%)
Mean estimated blood loss (mL)	117.0 ± 116.1
Mean operative time (min)	60.5 ± 30.8
Mean fluoroscopy time (sec)	271.4 ± 120.5

The cohort represented a broad spectrum of thoracic and thoracolumbar pathology, including thoracic degenerative disc disease, stenosis, myelopathy, kyphotic and scoliotic deformities, adjacent segment disease, trauma-related pathology, and revision surgery. The distribution of diagnostic categories is summarized in Table 2.

**Table 2:** Diagnostic categories in the study population (n=25)

Category	n (%)
Thoracic degenerative disease	13 (52.0)
Deformity/kyphosis/scoliosis	6 (24.0)
Stenosis/myelopathy	2 (8.0)
Other thoracic/thoracolumbar indications	4 (16.0)

Thoracic degenerative disease was the most common indication, accounting for 52.0% of cases (n=13), followed by deformity correction for kyphosis and/or scoliosis in 24.0% (n=6), and thoracic stenosis/myelopathy in 8.0% (n=2). The remaining cases reflected other thoracic and thoracolumbar indications, including revision and adjacent segment pathology.

The mean BMI for the entire cohort was 33.9 kg/m², reflecting a predominantly overweight and obese patient population. Six patients (24.0%) met the criteria for severe obesity (BMI ≥40 kg/m²), with BMI values extending as high as 51 kg/m². The series included both primary and revision procedures, isolated thoracic fusion procedures, multilevel thoracolumbar reconstructions, hybrid MIS-open constructs, and procedures combined with adjacent-level OLLIF techniques. Single-level MIS-OTIF was performed in 10 patients (40.0%), whereas 15 patients (60.0%) underwent multilevel procedures involving two or more thoracic levels. The most frequently treated levels included T10-T11, T11-T12, T10-T12, and T11-L1.

Across the entire cohort, mean EBL was 117.0 mL, mean operative time was 60.5 minutes, and mean fluoroscopy time was 271.4 seconds. Despite the complexity of many deformity, revision, and multilevel reconstructions, operative metrics remained favorable, supporting the efficiency and reproducibility of the MIS-OTIF technique.

Procedure characteristics

MIS-OTIF was successfully utilized both as a stand-alone thoracic fusion procedure and as a component of larger thoracolumbar reconstructive strategies. The distribution of surgical categories is presented in Table 3.

**Table 3:** Surgical categories MIS-OTIF: Minimally invasive oblique thoracic interbody fusion; OLLIF: oblique lateral lumbar interbody fusion.

Surgical Category	n (%)
Isolated MIS-OTIF	18 (72.0)
MIS-OTIF + OLLIF	6 (24.0)
MIS-OTIF + OLLIF + open posterior instrumentation	1 (4.0)
Total	25 (100)

Eighteen patients (72.0%) underwent isolated MIS-OTIF, six patients (24.0%) underwent combined MIS-OTIF and OLLIF procedures, and one patient (4.0%) underwent MIS-OTIF combined with OLLIF and open posterior instrumentation.

Representative combined constructs included T11-T12 MIS-OTIF with T12-L1 OLLIF, T11-T12 MIS-OTIF with T12-L3 OLLIF, T10-T12 MIS-OTIF with T12-L2 OLLIF, and T11-L1 MIS-OTIF combined with OLLIF. One patient underwent a complex hybrid reconstruction consisting of T10-T12 MIS-OTIF, T12-L1 OLLIF, and T9-L2 posterior instrumentation with selective skipped pedicle screw placement.

These findings demonstrate the versatility of MIS-OTIF as both a stand-alone thoracic fusion procedure and a complementary technique within extensive thoracolumbar reconstruction and deformity correction.

Operative time

Operative duration ranged from 30 to 171 minutes depending on procedural complexity. Single-level MIS-OTIF procedures demonstrated the shortest operative times, commonly ranging between 30 and 55 minutes (Table 4), which summarizes operative time, fluoroscopy time, and estimated blood loss according to surgical extent. 

**Table 4:** Perioperative metrics by surgical extent Data are presented as mean ± standard deviation (SD). Single-level procedures included one-level MIS-OTIF, whereas multilevel procedures included two or more instrumented levels.

Variable	Single-level (n=10)	Multilevel (n=15)
Blood loss (mL)	74.6 ± 49.4	145.3 ± 139.3
Operative time (min)	35.2 ± 7.9	77.4 ± 28.7
Fluoroscopy time (sec)	201.3 ± 57.0	318.1 ± 130.2

Longer operative durations were observed in revision procedures, reinstrumentation cases, multilevel thoracolumbar constructs, hybrid MIS-open reconstructions, and deformity corrections requiring supplemental fixation [2,4].

Comparative analysis demonstrated predictable increases in operative demands with increasing surgical complexity. Mean EBL increased from 74.6 mL in single-level procedures to 145.3 mL in multilevel procedures. Similarly, mean operative time increased from 35.2 minutes to 77.4 minutes, while mean fluoroscopy time increased from 201.3 seconds to 318.1 seconds. Compared with single-level procedures, multilevel MIS-OTIF was associated with a 95% increase in blood loss, a 120% increase in operative time, and a 58% increase in fluoroscopy utilization. These findings are consistent with the greater technical demands associated with multilevel thoracic reconstruction, including additional fluoroscopic localization, multiple cage placements, expanded instrumentation requirements, and deformity correction maneuvers.

BMI findings

The patient population demonstrated a broad BMI distribution ranging from normal BMI to severe obesity. Six patients (24.0%) were classified as having Class III obesity (BMI ≥40 kg/m²), with a mean BMI of 44.7 kg/m², while the remaining 19 patients (76.0%) had a mean BMI of 30.5 kg/m² (Table 5), which summarizes perioperative metrics by BMI category.

**Table 5:** Perioperative metrics by BMI category Data are presented as mean ± standard deviation. Statistical comparisons between the severe obesity and non-severe obesity groups were performed using an independent samples t-test (Welch's t-test for unequal variances). The test statistic (t) is reported alongside the corresponding p-values.

Variable	Severe obesity BMI ≥40 (n=6)	Non-severe obesity BMI <40 (n=19)	Test statistic (t)	p-value
Mean BMI (kg/m²)	44.3 ± 3.8	30.6 ± 4.0	7.60	<0.001
Mean estimated blood loss (mL)	108.8 ± 123.5	119.6 ± 117.1	−0.19	0.855
Mean operative time (min)	57.3 ± 48.3	61.5 ± 24.8	−0.20	0.845
Mean fluoroscopy time (sec)	178.3 ± 56.4	300.8 ± 121.2	−3.39	0.003

Fluoroscopy time is generally expected to increase in patients with severe obesity because increased soft-tissue thickness can reduce fluoroscopic image quality and necessitate additional imaging for accurate instrument localization and trajectory confirmation. The lower fluoroscopy time observed in the BMI ≥40 kg/m² group is likely explained by the predominance of single-level procedures within this subgroup, whereas the BMI <40 kg/m² cohort included a higher proportion of multilevel fusions requiring repeated fluoroscopic localization across multiple operative levels.

Despite the technical challenges traditionally associated with severe obesity, all patients with BMI ≥40 kg/m² successfully underwent MIS-OTIF without conversion to an open thoracic approach or obesity-related intraoperative complications. Mean EBL in this subgroup was 108.8 mL, mean operative time was 57.3 minutes, and mean fluoroscopy time was 178.3 seconds. These findings suggest that elevated BMI alone did not preclude efficient minimally invasive workflow and operative performance [6,15,17,19-21].

Compared with patients with BMI <40 kg/m², there were no statistically significant differences in estimated blood loss (108.8 vs. 119.6 mL, p=0.855) or operative time (57.3 vs. 61.5 minutes, p=0.845), indicating that severe obesity did not adversely affect operative efficiency. Although fluoroscopy utilization differed significantly between groups (178.3 vs. 300.8 seconds, p=0.003), this difference likely reflects procedural complexity rather than body habitus alone. The BMI <40 kg/m² cohort contained a greater proportion of multilevel procedures, which require additional fluoroscopic verification for level identification, trajectory planning, and implant positioning. Conversely, despite the expected challenges of increased soft-tissue depth in severely obese patients, MIS-OTIF remained technically feasible without increased operative time or blood loss.

Importantly, perioperative metrics in both BMI groups compared favorably with historical reports of open thoracic surgery. A retrospective review of open thoracic disc surgery reported a mean blood loss of 800 mL (range 400-1,500 mL), a mean operative time of 337.8 minutes (range 220-450 min), and a mean duration of hospitalization of 7.4 days, all of which are considerably higher than the values observed in the present MIS-OTIF cohort [22]. These findings suggest that MIS-OTIF may mitigate many of the perioperative burdens traditionally associated with thoracic spine surgery while maintaining favorable operative efficiency across a broad spectrum of patient body habitus.

Deformity cases

Six patients underwent MIS-OTIF primarily for correction of thoracic deformity (Table 6), including kyphosis and scoliosis.

**Table 6:** Cases of deformity treated with MIS-OTIF Construct level indicates the extent of the treated spinal segments. Associated procedures include adjunctive lumbar OLLIF and supplemental posterior instrumentation when performed; OLLIF: oblique lateral lumbar interbody fusion; MIS-OTIF: Minimally invasive trans-Kambin oblique thoracic interbody fusion.

Patient case	Deformity type	Construct level	MIS-OTIF construct	Associated procedures
Case 1	Kyphotic deformity	4 levels	T10–T12 MIS-OTIF	T12–L2 OLLIF
Case 2	Scoliosis	3 levels	T11–T12 MIS-OTIF	T12–L3 OLLIF
Case 3	Segmental scoliosis	5 levels	T10–T12 MIS-OTIF	T12–L1 OLLIF with T9–L2 posterior instrumentation
Case 4	Kyphotic deformity	4 levels	T8–T12 MIS-OTIF	—
Case 5	Scoliosis	3 levels	T10–L1 MIS-OTIF	T12–L1 OLLIF
Case 6	Scoliosis	3 levels	T11–L2 MIS-OTIF	T12–L2 OLLIF

These cases frequently involved multilevel constructs and commonly incorporated lumbar OLLIF procedures with supplemental posterior fixation. Representative constructs included T10-T12 MIS-OTIF with T12-L2 OLLIF, T11-T12 MIS-OTIF with T12-L3 OLLIF, and T10-T12 MIS-OTIF with T12-L1 OLLIF combined with T9-L2 posterior instrumentation.

The successful completion of these complex reconstructions without conversion to open thoracic exposure highlights the adaptability of MIS-OTIF beyond isolated degenerative pathology and supports its application in carefully selected deformity patients.

Hospital length of stay

Hospital length of stay data were available for all 25 patients (Table 7).

**Table 7:** Hospital length of stay following MIS-OTIF Hospital length of stay was assessed in all patients undergoing minimally invasive trans-Kambin oblique thoracic interbody fusion (MIS-OTIF). Values represent the number and percentage of patients discharged at each postoperative day.

Hospital length of stay (days)	Number of patients (n)	Percentage (%)
0 days (same-day discharge)	4	16.0%
1 day	18	72.0%
2 days	1	4.0%
3 days	2	8.0%
Total	25	100%

The mean hospital stay was 1.04 days (range, zero to three days). Eighteen patients (72.0%) were discharged on postoperative day one, while four patients (16.0%) were discharged on the day of surgery and three patients (12.0%) required hospitalization beyond one postoperative day. The short duration of hospitalization observed in this cohort reflects the minimally invasive nature of the MIS-OTIF technique and supports its association with expedited postoperative recovery and early mobilization.

Safety analysis and complications

Overall complication rates remained low. Persistent residual stenosis was identified in one patient and represented the only notable postoperative complication within the reviewed cohort. No intraoperative neurological injuries, vascular injuries, deep wound infections, or catastrophic approach-related complications were identified [8]. These results are summarized in Table 8.

**Table 8:** Complications and safety outcomes

Outcome	n (%)
Persistent residual stenosis	1 (4.0)
Neurological injury	0 (0)
Major vascular injury	0 (0)
Deep wound infection	0 (0)
Pneumothorax	0 (0)
Conversion to open thoracic approach	0 (0)

Notably, no pneumothorax occurred in this series, a finding of particular importance given that pleural violation and pneumothorax remain recognized risks of transthoracic approaches and certain minimally invasive thoracic fusion techniques, including MIS-DTIF [18]. Furthermore, no case required conversion to an open thoracic approach, and MIS-OTIF was successfully completed in all 25 patients. Taken together, these findings demonstrate a favorable safety profile, high procedural completion rate, and reproducible operative performance across a heterogeneous patient population encompassing degenerative disease, deformity, revision surgery, obesity, and multilevel thoracolumbar reconstruction. The absence of major approach-related morbidity supports MIS-OTIF as a safe and technically feasible alternative to conventional thoracic fusion approaches.

## Discussion

The present study demonstrates that MIS-OTIF can maintain efficient operative workflow even in technically demanding thoracolumbar cases. Operative time efficiency is increasingly recognized as a critical determinant of perioperative outcomes, anesthesia exposure, resource utilization, blood loss, and surgeon fatigue [2,4].

Traditional open thoracolumbar fusion frequently requires extensive subcostal dissection, wide muscular stripping, prolonged exposure, significant blood loss, and prolonged closure time [1-3].

By contrast, MIS-OTIF minimizes tissue disruption through targeted access corridors and fluoroscopically guided instrumentation. Potential contributors to operative efficiency include smaller incisions, reduced exposure requirements, streamlined fluoroscopic targeting, percutaneous instrumentation, limited muscle dissection, and reduced closure burden [5,6].

Obesity remains a major challenge in spine surgery. Open thoracolumbar procedures in obese patients are frequently associated with increased operative duration, greater wound complications, higher blood loss, difficult exposure, and increased physiologic stress [6,15].

In the present cohort, elevated BMI did not independently prohibit efficient minimally invasive completion. Several morbidly obese patients underwent successful MIS-OTIF procedures while maintaining acceptable operative duration. This finding suggests that minimally invasive oblique approaches may significantly mitigate the exposure-related challenges encountered during traditional open surgery in obese patients [6].

Minimally invasive thoracolumbar fusion techniques have consistently demonstrated reduced blood loss compared with open surgery in prior literature [1,3]. Reduced muscular dissection, limited epidural venous disruption, smaller operative corridors, and reduced dead space formation will contribute to lower transfusion requirements and earlier mobilization [3,17]. Furthermore, historical evaluations of classic minimally invasive trans-Kambin approach demonstrate an early learning curve that stabilizes significantly after initial operative sets, decreasing blood loss and recovery times dynamically [19-21]. In fact, none of the patients in our series required blood transfusions.

Hybrid constructs and revision procedures predictably demonstrated longer operative duration. Prior surgery results in altered tissue planes, scar formation, more difficult fluoroscopic interpretation, and increased instrumentation complexity [13].

Despite these challenges, MIS-OTIF remained technically feasible and operative times remained superior relative to traditional open thoracolumbar revision.

Thoracic spinal procedures carry increased neurologic risk due to proximity of the spinal cord and narrow canal dimensions. Careful biplanar fluoroscopic targeting in MIS-OTIF, pedicle trajectory planning, and minimally disruptive access are essential for safe execution [14,16].

Limitations

This study has several limitations. First, the retrospective design and relatively small sample size of 25 patients limit the generalizability of the findings. Additionally, all procedures were performed by a single experienced surgeon, which may introduce surgeon-specific bias and limit the applicability of the results across different practice settings. MIS-OTIF is a technically demanding procedure with a learning curve; therefore, further studies involving multiple surgeons with varying levels of experience and larger patient cohorts are needed to validate these findings, assess reproducibility, and better define the broader clinical applicability and safety profile of this technique. Future multicenter prospective studies will be valuable in evaluating outcomes across diverse surgical environments and patient populations.

Future directions

Future studies should focus on further defining the long-term clinical utility and broader applicability of MIS-OTIF through prospective, multicenter investigations. Important areas of future research include evaluation of long-term fusion rates, patient-reported outcomes, functional recovery, perioperative blood loss, radiation exposure, and detailed learning curve analysis. Additional studies assessing cost-effectiveness, the role of navigation-assisted and robotic-assisted instrumentation, and direct comparisons with traditional open thoracolumbar fusion techniques will be essential to establish the relative advantages, limitations, and optimal indications for MIS-OTIF. These investigations will help refine surgical strategies, improve reproducibility, and support evidence-based adoption of this minimally invasive approach.

## Conclusions

MIS-OTIF demonstrates favorable operative efficiency across a broad spectrum of thoracic and thoracolumbar pathology, including obese patients, multilevel constructs, and revision surgery. The minimally invasive oblique corridor may contribute to reduced operative exposure, decreased paraspinal muscular disruption, acceptable operative time, reduced physiologic stress response, lower intraoperative blood loss, shorter hospital length of stay, and improved perioperative workflow efficiency.

Clinical outcomes within this cohort demonstrated rapid postoperative recovery, with many patients achieving same-day discharge, the majority discharged within postoperative day one, and a maximum hospital stay of three days, supporting the potential benefits of minimally invasive thoracolumbar access techniques in reducing inpatient utilization and accelerating recovery pathways.

Hybrid and revision procedures remain technically more demanding and are associated with prolonged operative duration; however, minimally invasive surgical principles continue to provide meaningful workflow and recovery advantages even in complex cases. Further multicenter prospective investigations with larger patient populations and long-term clinical follow-up are necessary to validate these findings, assess long-term functional and radiographic outcomes, and establish standardized indications for MIS-OTIF.
